# A Self-Powered Biosensor for Monitoring Maximal Lactate Steady State in Sport Training

**DOI:** 10.3390/bios10070075

**Published:** 2020-07-08

**Authors:** Yupeng Mao, Wen Yue, Tianming Zhao, MaiLun Shen, Bing Liu, Song Chen

**Affiliations:** 1 Physical Education Department, Northeastern University, Shenyang 110819, China; maoyupeng@pe.neu.edu.cn (Y.M.); sml1995@163.com (M.S.); 2 Department of Physical Education, Beihang University, Beijing 100191, China; sqxs9@buaa.edu.cn; 3 College of Sciences, Northeastern University, Shenyang 110819, China; zhaotm@stumail.neu.edu.cn; 4 School of Arts, Beijing Sport University, Beijing 100084, China; zhaochongle@pe.neu.edu.cn

**Keywords:** biosensor, T-ZnO, self-powered, maximal lactate steady state

## Abstract

A self-powered biosensor for monitoring the maximal lactate steady state (MLSS) during exercise has been developed for intelligently assisting training system. It has been presented to create poly (vinylidene fluoride) (PVDF)/Tetrapod-shaped ZnO (T-ZnO)/enzyme-modified nanocomposite film through an efficient and cost-effective fabrication process. This sensor can be readily attached to the skin surface of the tester. Due to the piezoelectric surface coupling effect, this biosensor can monitor/sense and analyze physical information in real-time under the non-invasive condition and work independently without any battery. By actively outputting piezoelectric signals, it can quickly and sensitively detect body movements (changes of joint angle, frequency relative humidity during exercise) and physiological information (changes of lactate concentration in sweat). A practical application has been demonstrated by an excellent professional speed skater (male). The purpose of this study is to increase the efficiency of MLSS evaluation, promote the development of piezoelectric surface coupling effect and motion monitoring application, develop an intelligently assisting training system, which has opened up a new direction for human motion monitoring.

## 1. Introduction

In recent years, many researchers have attempted to combine the human body with various sensors to capture the information of body movements and physiological indexes [1,2,3,4,5,6]. These sensors can detect temperature, heart rate, and breath, which can provide convenience of health care monitoring and medical diagnosis [7,8,9,10,11,12].

And some commercial sensors have been applied to sport monitoring technology to monitor athletes’ aerobic ability and make scientific and effective training plans [13,14,15,16,17,18]. The maximal lactate steady state (MLSS) is the gold standard to evaluate aerobic (endurance) ability, which reflects the higher the MLSS is, the higher aerobic ability the athlete has, and it is related to the way of energy supply [19,20,21,22,23,24]. However, there are some problems to be resolved in the evaluation process. First, in traditional test, the maximal oxygen consumption and blood lactate value should be collected for comprehensive evaluation, the collection process not only needs to interrupt the movement process of the subjects, but also increases the risk of infection by damaging the skin surface [21,22,24]. Secondly, the entire evaluation process requires the cooperation of multiple devices, multiple units cannot be integrated, traditional power supply and battery are indispensable, maintenance is relatively troublesome, service life may be short, and equipment is expensive. Therefore, it is important to design a kind of flexible biosensor with real-time response and without power unit. poly (vinylidene fluoride) (PVDF) and T-ZnO have been regarded as a candidate for novel biosensing material, due to the excellent piezoelectric property, sensitive sensing characteristics, and good biological compatibility [25,26,27,28,29,30,31,32]. Through the piezoelectric effect, the material can transfer tiny mechanical energy into electric energy. The piezoelectric output increases with the applied force. However, the architecture and material of the biosensor need to be further explored to realize MLSS detecting in real-time.

In this paper, a multifunctional, flexible, and portable biosensor based on PVDF/T-ZnO/enzyme-modified nanocomposite film has been constructed to monitor the maximal lactate steady state (MLSS) during exercise. The sensor can be readily attached to the skin surface of the tester. Through modifying enzyme on the surface of the PVDF/T-ZnO composite film, the biosensor can monitor/sense and analyze physical information in real-time under the non-invasive condition and work independently without any power units. Due to the piezoelectric surface coupling effect. It can quickly and sensitively detect body movements (changes of joint angle, frequency, and relative humidity during exercise) and physiological information (changes of lactate concentration in sweat). An excellent professional speed skater (male) is invited to verify the practical application, showing the feasibility of monitoring the MLSS in the future. The purpose of this study is to increase the efficiency of MLSS evaluation, promote the development of piezoelectric surface coupling effect and motion monitoring application, develop the intelligently assisting training system, which has opened up a new direction for human motion monitoring.

## 2. Materials and Methods

### 2.1. Synthesis of T-ZnO on Textiles

The T-ZnO (purchased from Chengdu Tianyou Jingchuang Technology, Chengdu, China) were firmly fixed on the textiles (various woven fabrics) by a simple wet using PVDF binder. Firstly, the fabric was washed several times with pure water and ethanol. Secondly, it was treated in an ultrasonic bath for 10 min to remove surface impurities and dried in air at 60 °C Then, 2.5 g PVDF powder was dissolved in 50 mL acetone solution and stirred at 60 °C for 1 h. Thirdly, 5 g of T-Zno nanostructures were added to PVDF gel and stirred at 60 °C for 1h. Finally, uniform T-ZnO/PVDF paste was applied to the washed fabric (14 × 14 cm) and dried in air at 60 °C. Moreover, the size can be cut according to the actual application.

### 2.2. Fabrication of Self-Powered Device

Firstly, the fabric was cleaned through the above steps. Then the copper foil was used as two electrodes to attach both sides of prepared fabric. Secondly, Kapton was invoked as the flexible substrate to support the T-ZnO nanostructures fabric device. Thirdly, T-ZnO were modified with lacticoxidase (LOx) provided by Sigma Chemical Co., LTD, Shanghai, China. 0.5 mL (10 g/L) LOx solution was slowly dropped onto the surface of T-ZnO nanowire arrays and naturally dried for 2–3 h. This procedure was replicated for 4 times to make LOx fully attached.

### 2.3. Measurement

The morphology and structure of nanowire arrays and devices were studied by scanning electron microscopy (SEM, Hitachi s4800, Tokyo, Japan). The low noise preamplifier (Stanford Research System SR560, Guangdong, China) was used to measure the piezoelectric output. Lactate scout-KEF was used to measure blood lactate concentration. The performance of the sensor was tested with the measurement system (a stepper motor, a slide way and supports). This system can provide the pre-set driving force. The maximal lactate steady state was determined by the traditional evaluation method (Monark Ergomedic 839E, Stockholm, Sweden, combined with maximal oxygen consumption) within 96 h before the test. The device was attached to the tester for practical verification and application test. Unless particularly stated, all experimental measurements were at relative humidity (25%) and room temperature (25 °C). The subject had been given his informed consent for inclusion before they participated in the study. All subjects gave their informed consent for inclusion before they participated in the study. The study was conducted in accordance with the Declaration of Helsinki, and the protocol was approved by the Ethics Committee.

## 3. Results

**Figure 1:**
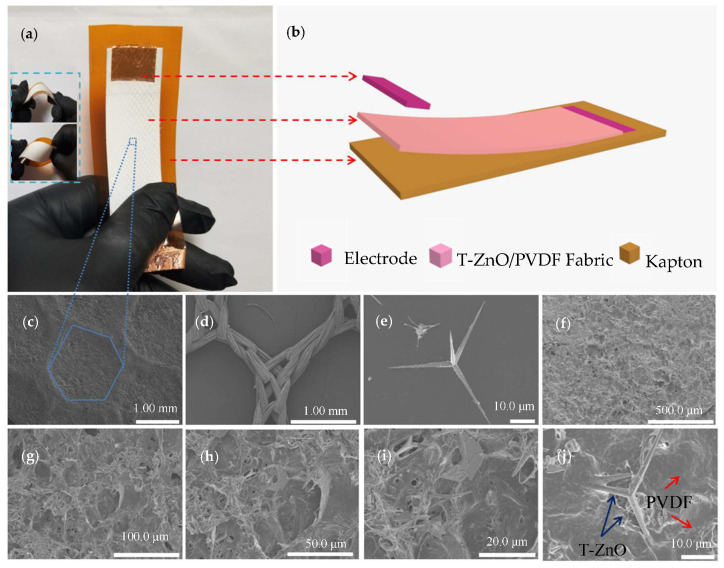
Structure of self-powered biosensor. (**a**) Optical image of the device. (**b**) Device structure. (**c**) SEM image of T-ZnO/poly (vinylidene fluoride) (PVDF)/fabric. (**d**) SEM image of organic fabric. (**e**) SEM image of one single T-ZnO. (**f**–**j**) High-power SEM images of T-ZnO/PVDF/fabric with different magnification.

The optical image, sensor structure and SEM images of the self-powered biosensor are shown in Figure 1. Figure 1a shows the optical image of the sensor, which can be shaped at will (bending, twisting) and cut into any shapes to meet the test requirements. Figure 1b shows the device structure of the device. T-ZnO, when mixed with acetone and PVDF, are uniformly and firmly fixed on the organic fabric. At this time, the T-ZnO with three-dimensional structure have formed a nano array [33,34]. The copper foils on the upper and lower sides serve as electrodes, and the flexible Kapton plate is used as the support. Figure 1c shows the SEM image of T-ZnO/PVDF/fabric, and it can be clearly observed the fabric profile. The organic fabric is shown in Figure 1d. Figure 1e shows the SEM image of one single T-ZnO. The morphology of nanowire arrays growing in four directions and the overall morphology of T-ZnO can be observed. Figure 1h–j shows different magnification of high-power SEM images of T-ZnO/PVDF/fabric. Some optical photographs are shown in Figure S1 (Supplementary Materials). The T-ZnO is fixed on the PVDF/fabric surface to form nanowire arrays structure.

**Figure 2:**
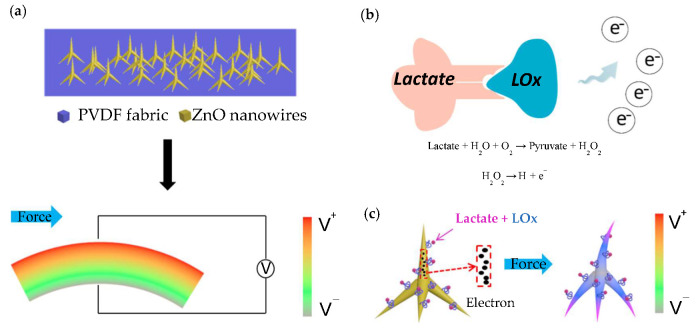
A working mechanism sketch of self-powered biosensor. (**a**) Power generation mechanism of T-ZnO/PVDF/fabric. (**b**) Enzymatic reaction. (**c**) Piezoelectric surface coupling effect.

A working mechanism sketch (not corresponding to actual measurements) of self-powered biosensor has been shown in Figure 2. Figure 2a shows the power generation mechanism of T-ZnO/PVDF/fabric. At the beginning, the device has no piezoelectric output without applying external force. When it is deformed by external force, the PVDF film wrapped around T-ZnO generates an electric field on the stretching and compressing surface during deformation, and outputs piezoelectric voltage signal due to the piezoelectric effect [35]. The color legend of the rainbow presents the magnitude of the outputting piezoelectric voltage. The piezoelectric output of T-ZnO/PVDF nanocomposite film relies on the mechanical energy provided by the external force (monitoring object). Therefore, the piezoelectric can be regarded as a kind of signal of pressure. The whole sensing process does not need any external power supply. The enzymatic reaction of lactate and lacticoxidase (LOx) has been shown in Figure 2b. When lactate contacts with the enzyme, enzymatic reaction occurs, pyruvate and H_2_O_2_ will be generated first as follows [36]:

(1) $$Lactate+H_{2}O+O_{2}\overset{LO_{x}}{\to }\;Pyruvate+H_{2}O_{2}$$

The H_2_O_2_ is unstable, some of them existing in the form of H^+^, O_2_ and e^−^. The decomposition formula is as follows [37]:

(2) $$H_{2}O_{2}\;\to \;2H^{+}+O_{2}+2e^{-}$$

Figure 2c shows the piezoelectric surface coupling effect (coupling with piezoelectric effect and enzymatic reaction). ZnO has an excellent property of biological compatibility and the enzyme molecules can be co-deposited with organic matter and fixed on the surface of the T-ZnO nanowire arrays [30,38,39]. The enzymatic reaction takes places at the interface of ZnO and LOx. It has been reported that H_2_O_2_ can transfer electrons to T-ZnO nanowire arrays by producing H^+^ and e^−^ and increase the surface carrier density [30,36,40,41]. In this process, H^+^ ions are adsorbed on the surface of T-ZnO nanowire arrays as additional carriers which enhances the shielding effect. When external force deformation is applied, the piezoelectric output of T-ZnO nanowire arrays is reduced due to the strong piezoelectric shielding effect.

**Figure 3:**
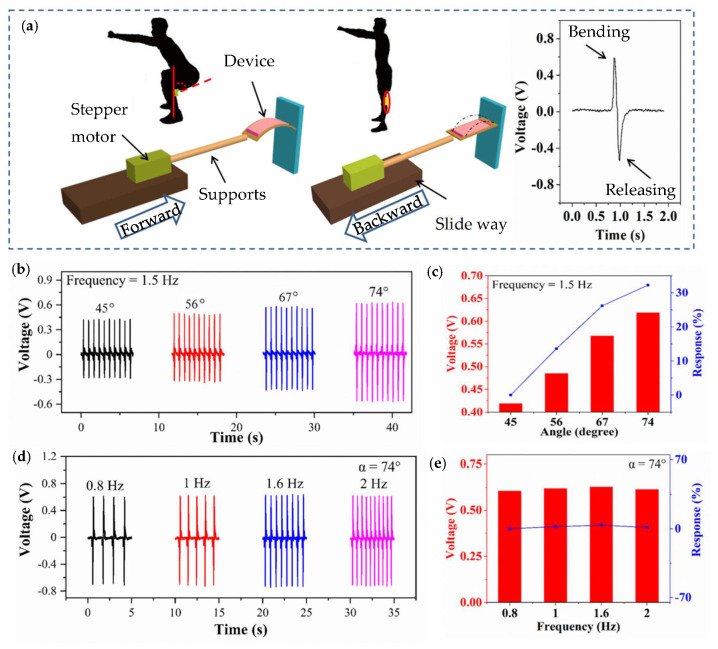
Biosensor for detecting angle and frequency in real-time. (**a**) Measurement system. The inset shows the outputting piezoelectric voltage of one cycle. (**b**) Outputting piezoelectric voltage of the sensor against different angles (1.5 Hz). (**c**) The relationship between angle and outputting piezoelectric voltage. (**d**) Outputting piezoelectric voltage of the sensor against different frequency (74°). (**e**) The relationship between the frequency and outputting piezoelectric voltage.

Figure 3 shows the self-powered biosensor for detecting angle and frequency in real-time. Figure 3a shows the measurement system, which aims to mimic the movements of joints. The inset shows the outputting piezoelectric voltage of one cycle. The optical photograph of this measurement system is shown in Figure S2 (Supplementary Materials). The outputting piezoelectric voltage of different mass fraction of T-ZnO is shown in Figure S3 (Supplementary Materials). When the mass fraction of T-ZnO is 66.7%, the device will output stable piezoelectric voltage. The little outputting piezoelectric voltage of pure PVDF is due to lack of the polarization treatment. The more T-ZnO leads to destroy the flexibility of this device. In order to assist the experiment with more accurate angle and frequency under control, the deformation is provided from a programmable measurement system. Figure 3b shows the outputting piezoelectric voltage of the biosensor against different angles (1.5 Hz). The outputting piezoelectric voltage of the device increases with the bending angle. When the bending angle is 45, 56, 67, and 74°, the outputting piezoelectric voltage of the device is 0.419, 0.485, 0.567, and 0.619 V, respectively. Figure 3c shows the relationship between the angle and the outputting piezoelectric voltage. The response of the device can be calculated from the following equation:

(3) $$R\%=\left|\frac{V_{0}-V_{i}}{V_{i}}\right|\times 100\%$$

where V_0_ and V_i_ are the outputting piezoelectric voltage of 45° and other angles, respectively. When the bending angle of the device is 45, 56, 67 and 74°, the response is 0%, 13.6%, 26.2%, and 23.2%, respectively. Figure 3d shows the outputting piezoelectric voltage against different frequencies (74°). When the deformation angle of the sensor is constant and the frequency is altered, the outputting piezoelectric voltage is almost a constant (Figure 3e). When the deformation frequency of the sensor is 0.8, 1, 1.6, and 2 Hz, the response of the piezoelectric voltage is 0%, 2%, 3%, and 1%, respectively. The above evidence demonstrates that the outputting piezoelectric voltage of biosensor mainly depends on the degree of deformation and the effect of frequency transformation on the piezoelectric voltage output can be ignored. Furthermore, the self-powered sensor can be driven by body movements and actively output sensing signal. The whole process does not need any external power supplies. This property can be implemented to the monitoring of joint angle change and motion frequency of the trunk during human movement. It can help the disabled or the central nervous system injured to carry on the sport rehabilitation evaluation monitoring [18,30,36] and monitor the high-level athlete training program to improve the sport performance [13,19,20,21,42].

**Figure 4:**
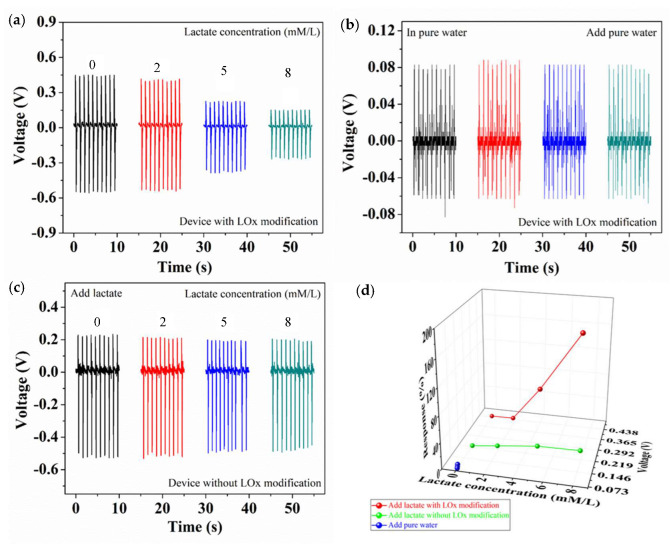
The biosensor performance for detecting lactate concentration (**a**) The outputting piezoelectric voltage of the biosensor (modified with lacticoxidase (LOx)) against different lactate concentration from 0 to 8 mmol/L (45°). (**b**) The outputting piezoelectric voltage of the biosensor (modified LOx) in pure water (45°). (**c**) Outputting piezoelectric voltage of the biosensor (unmodified with LOx) against different lactate concentration from 0 to 8 mmol/L. (**d**) The outputting piezoelectric voltage and response of the three devices.

The biosensor performance for detecting lactate concentration is shown in Figure 4. The target solution (0.5 mL) was dropped on the test device, and the device is driven by the programmable measurement system (45°). Generated the piezoelectric voltage signal (Both as a power source and as a biosensor signal) through the piezoelectric surface coupling effect, the piezoelectric voltage signal can act as both a power source and a biosensor signal [43,44,45]. Figure 4a shows that the outputting piezoelectric voltage of the device decreases with the increasing in the lactate concentration. When the lactate concentration is 0, 2, 5, and 8 mmol/L, the outputting piezoelectric voltage of the device is 0.446, 0.404, 0.221, and 0.150 V, respectively. In order to confirm the piezoelectric surface coupling effect, two control groups have been designed as shown in Figure 4b,c. Figure 4b shows that the target solution is pure water, and the outputting piezoelectric voltage of the device with LOx modification is 0.081, 0.085, 0.083, and 0.080 V, respectively. Figure 4c shows that the outputting piezoelectric voltage of the device without LOx modification is 0.225, 0.21, 0.195, and 0.197 V, respectively. As a result, lower outputting piezoelectric voltage is contributed to the piezoelectric surface coupling effect. Figure 4d shows the outputting piezoelectric voltage and response of three devices. When the target solution of pure water is dripped on the device with LOx modification and the lactate solution of 0, 2, 5, and 8 mmol/L is dripped on the device without LOx modification, the response is not obvious. As the concentration of lactate is 0, 2, 5, and 8 mmol/L, the response is 0%, 10%, 100%, and 197%, respectively. This evidence indicates that the decreasing in the outputting piezoelectric voltage of the device with LOx modification goes hand in hand with the increasing in concentration of lactate, which is owed to the piezoelectric surface effect.

**Figure 5:**
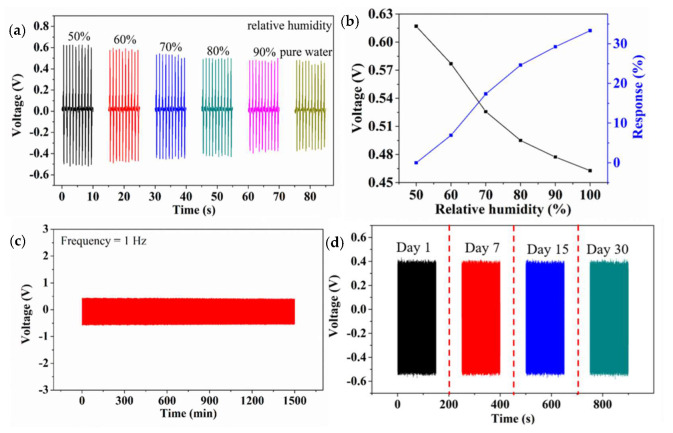
(**a**) The outputting piezoelectric voltage of biosensor against different relative humidity. (**b**) The outputting piezoelectric voltage and response of the biosensor against different relative humidity. (**c**) The stability of the biosensor. (**d**) The lifetime of the biosensor.

In the process of exercise, a large number of skeletal muscles participate in the work to generate heat, and the skin perspires in the temperature balance. So, the influence of relative humidity to the biosensor needs to be investigated. As shown in Figure 5a, the outputting piezoelectric voltage of biosensor decreases with the increasing relative humidity, until the relative humidity reaches 100% (pure water). Figure 5b shows the outputting piezoelectric voltage and response of the biosensor against different relative humidity. When the device is exposed to a humid environment, the water molecules will quickly occupy the available sites. The dissociative adsorption of the water releases electrons and protons to strong the piezo-screening effect, leading to the lower outputting piezoelectric voltage [46,47,48]. When the relative humidity is 50%, 60%, 70%, 80%, 90%, and 100% (pure water), the response of the device is 0%, 6%, 17.3%, 24.6%, 29.2%, and 33.3%, respectively. Figure 5c shows in the stability of the biosensor after continuous working for 1500 min. After 30 days, the outputting piezoelectric voltage is pretty much constant as shown in Figure 5d. It can be seen that the biosensor has good stability and repeatability and can meet the requirements of long-term sport training monitoring in real-time.

**Figure 6:**
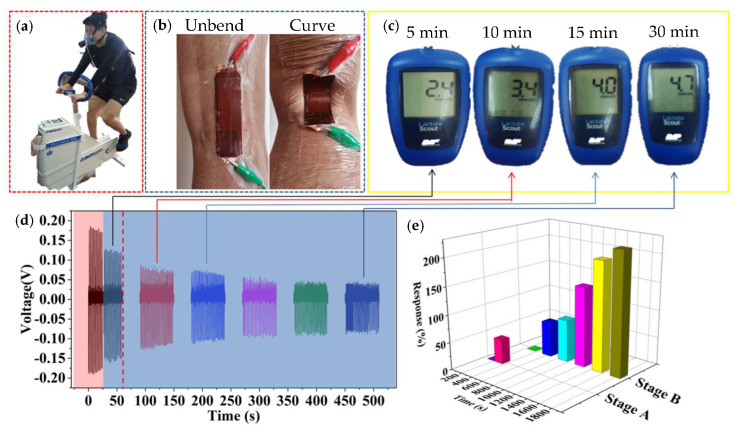
The practical application of biosensor. (**a**) Optical image of subject power bicycle tests. (**b**) The optical image of the joint position (stretching and bending) with the biosensor. (**c**) Blood lactate concentration of tester at different time points by the commercial sensor. (**d**) The real-time outputting piezoelectric voltage of biosensor. (**e**) The real-time response of biosensor.

Figure 6 shows the practical application of biosensors for monitoring and verifying the MLSS. The tester is an excellent professional speed skater (male) and did not exercise or drink alcohol or caffeine for at least 24 h before this test. The power bike (Monark Ergomedic 839E, Stockholm, Sweden) integrates with a commercial sensor. Our biosensor is attached to the knee-joint, as shown in Figure 6a,b. The MLSS is 5 ± 0.5 mmol when the resistance of the power bicycle is 100 W and the speed is 60 rpm for 30 min at a constant pedal speed. Figure 6c shows that the blood lactate concentrations of the athlete are 2.4, 3.4, 4.0, and 4.7 mmol/L at the 5, 10, 15, and 30th min, respectively. Figure 6d shows the piezoelectric voltage output of the biosensor at the 5, 10, 15, 20, 25, and 30th min. It should be pointed out that the abrupt change of the fifth minute (red area) is due to the sweat. After perspiring (blue area), the outputting piezoelectric voltage of our biosensor is dependent on the lactate concentration. As blood lactate value (collect fingertip blood) is 2.4, 3.4, 4.0, and 4.7 mmol, the output piezoelectric voltage is 0.122, 0.074, 0.069, and 0.038 V, respectively. Through outputting piezoelectric voltage of biosensor, it can be observed that the piezoelectric output tends to be steady after 15 min. This result conforms to the commercial sensor and this physiological phenomenon conforms to the range of MLSS (±1 mmol) [41,42,49]. Figure 6e shows the response of the biosensor during the test. Stage A is the response of the biosensor in the stage from non-sweat to sweat due to the change in relative humidity. When the piezoelectric voltage is 0.177 and 0.122 V, the response is 0% and 45%, respectively. Stage B is the response of biosensor for detecting lactate concentration of sweat. When the voltage is 0.122, 0.074, 0.069, 0.05, 0.041, and 0.038 V, the response is 0%, 63.9%, 77.2%, 144.8%, 197.4%, and 220.8%, respectively. These results show that it is feasible to monitor the MLSS in our biosensor. So, a multifunctional, flexible, portable, non-invasive, and self-powered biosensor based on PVDF/T-ZnO/Lox-modified nanocomposite film has been constructed to monitor the MLSS during exercise. Although the biosensor is not yet up to industry standards, it is hoped that the concept of portable biosensors will help many people in the need to formulate exercise prescriptions through further development in the future.

## 4. Conclusions

To sum up, a multifunctional, flexible, and portable biosensor based on PVDF/T-ZnO/enzyme-modified nanocomposite film has been presented to monitor the MLSS in the process of movement. Due to the piezoelectric surface coupling effect, the piezoelectric output can be regarded as biosensing signals. The flexible biosensor can quickly and sensitively detect the changes in joint angle, frequency, relative humidity, and physiological information during movement. Under the non-invasive condition, real-time monitoring/analyzing is carried out for the training process of athletes and help to formulate a scientific and effective exercise prescription for special groups. This multidisciplinary research may offer new ideas for the development of sport medicine and wearable devices.

## Supplementary Materials

The following are available online at https://www.mdpi.com/2079-6374/10/7/75/s1, Figure S1: Optical photographs of self-powered biosensor, Figure S2: Optical photograph of the measurement system. Figure S3. The outputting piezoelectric voltage of different mass fraction of T-ZnO.
