# Supplementary material for: A Self-Powered Biosensor for Monitoring Maximal Lactate Steady State in Sport Training
Source: Biosensors (Basel). 2020 Jul 8;10(7):75. doi: 10.3390/bios10070075 (PMC7399796; doi:10.3390/bios10070075)
Supplement: Supplementary file 1 [file biosensors-10-00075-s001.pdf]

biosensors

Article

A Self-Powered Biosensor for Monitoring Maximal Lactate Steady State in Sport Training

Yupeng Mao [1,†], Wen Yue [2,†], Tianming Zhao [3], MaiLun Shen [1], Bing Liu [4] and Song Chen [1,*]

1 Physical Education Department, Northeastern University, Shenyang 110819, China; maoyupeng@pe.neu.edu.cn (Y.M.); sml1995@163.com (M.S.)
2 Department of Physical Education, Beihang University, Beijing 100191, China; sqxs9@buaa.edu.cn
3 College of Sciences, Northeastern University, Shenyang 110819, China; zhaotm@stumail.neu.edu.cn
4 School of Arts, Beijing Sport University, Beijing 100084, China; zhaochongle@pe.neu.edu.cn
* Correspondence: chensong@pe.neu.edu.cn
† These two authors contributed equally to this work.

Received: 3 June 2020; Accepted: 3 July 2020; Published: 8 July 2020

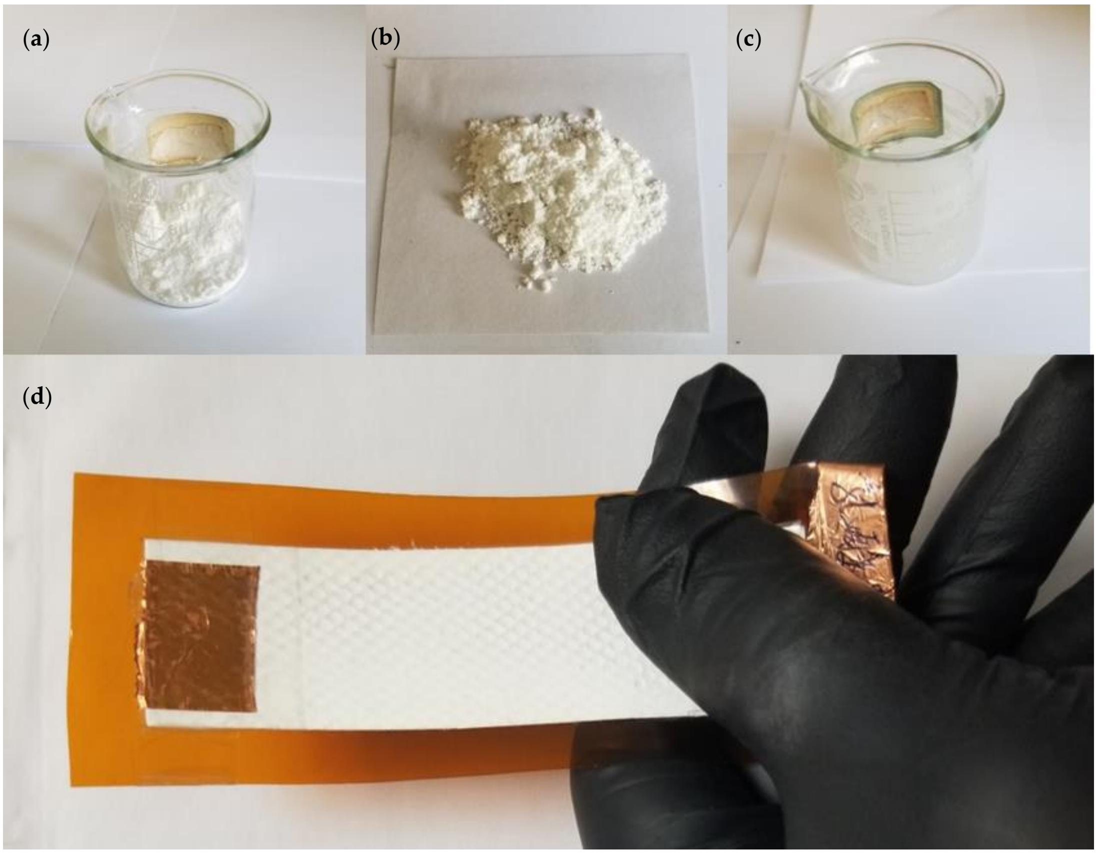

Figure S1. Optical photographs of self-powered biosensor. (**a**) PVDF (**b**)T-ZnO (**c**) T-ZnO/PVDF paste (**d**) self-powered device.

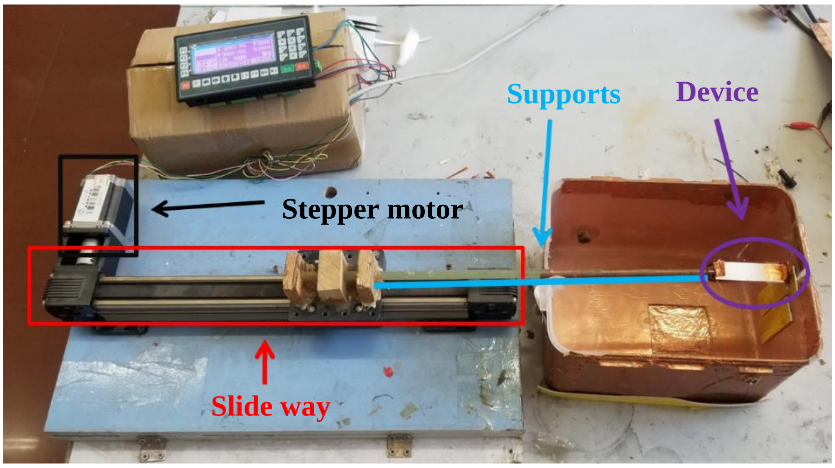

Figure S2. Optical photograph of the measurement system.

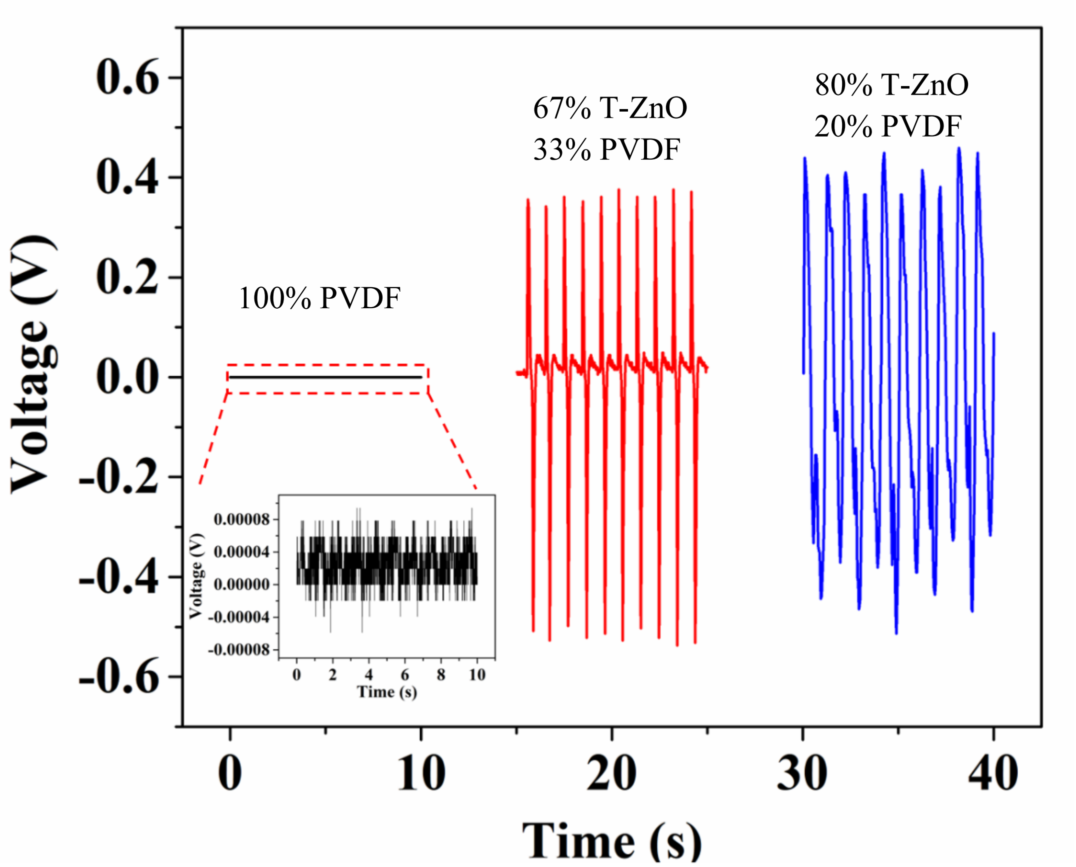

Figure S3. The outputting piezoelectric voltage of different mass fraction of T-ZnO.
